# Embryotoxic and Teratogenic Effects of Nickel in Swiss Albino Mice during Organogenetic Period

**DOI:** 10.1155/2013/701439

**Published:** 2013-07-15

**Authors:** Shivi Saini, Neena Nair, Mali Ram Saini

**Affiliations:** ^1^Cell and Molecular Biology Laboratory, Department of Zoology, University of Rajasthan, Jaipur, Rajasthan 302055, India; ^2^Radiation and Cancer Biology Laboratory, Department of Zoology, University of Rajasthan, Jaipur, Rajasthan, India

## Abstract

The present study evaluates potential hazardous of nickel (Ni^+2^ as NiCl_2_ *·*6H_2_O) to Swiss albino mice fetus. Ni was administered orally on body weight base from days 6 to 13 of gestation period. Based on LD_50_, Ni doses (46.125, 92.25, and 184.5) mg Ni/kg b.wt. were used. On day 18 of gestation, uteri of the sacrificed dams were examined. A dose-dependent decrease (*P* < 0.01) in the body weight of the pregnant females and fetuses during the gestation period was observed. Number of implant sites and placental weight at all the three dose levels was lower compared with their respective control groups. Average number of live fetuses/dams reduced significantly (*P* < 0.01) at 184.5 mg Ni/kg b.wt. with concomitant increase in the percentage of postimplantation death and percentage of resorbed, macerated, and dead fetuses, respectively. Exposure increased the fetal malformations, namely, hydrocephaly, open eyelids, microphthalmia, exophthalmia, club foot, umbilical hernia, and skeletal anomalies. Reduced ossification of nasal, frontal, parietal, intraparietal, and supraoccipital bones, absence/gap between the ribs, reduced/fused sternebrae, vertebral centra, and caudal vertebrae, reduced pelvic elements, absence of carpals, metacarpals, tarsals, metatarsals, and phalanges were distinct. This indicates vulnerability of the mice fetus to nickel during prenatal exposure.

## 1. Introduction

Human beings and wild life are constantly exposed to environmental contaminants. Nickel widely used in industries for various processes such as catalysts, dye mordant, electroplating, and so forth [1] has become a serious problem throughout the world. Although exposure to nickel can occur through percutaneous absorption or inhalation (occupational exposure) or ingestion via food and drinking water [2], but primarily it is through contaminated drinking water due to seepage in the underground water around the work place and also from food [3]. Nickel is the most common cause of allergy in the form of contact dermatitis [4] and hand eczema [5]. Lung fibrosis, cardiovascular disease, kidney diseases, asthma, inflammatory reactions, and hematopoiesis have been reported on exposure to nickel compounds [6–8].

A case report of occupational exposure of Russian women to nickel hydrometallurgy refinery plant resulted in complications during pregnancy with high incidence of spontaneous and threatening abortions, congenital malformations, and cardiovascular and musculoskeletal defects [9–12]. Soluble or insoluble nickel salts have been reported to have effects on developmental reproductive systems in animals [7, 8, 13, 14]. Increased number of resorptions as well as fetal mortality in pregnant golden hamsters after i.p. administration [15] and i.v. administration in ICR mice [16] has been observed. Embryotoxicity and fetotoxicity have also been reported using nickel salts [17]. Keeping in view the adverse impact of Ni upon human health, a study was planned to evaluate the embryotoxic and teratogenic potentials of Ni^+2^ as NiCl_2_·6H_2_O during organogenetic period in Swiss albino mice.

## 2. Materials and Methods

### 2.1. Animals

Swiss albino mice (7–9 weeks of age, 24 ± 2 gm) selected from an inbred colony were maintained on standard mice feed (Aashirwad Ltd., Chandigarh) and tap water *ad libitum*. Female and male mice were housed for mating in the ratio of 3 : 1 and examined every morning for vaginal plug. The day on which vaginal plug were detected was considered as day zero of pregnancy. The experiments was approved by the Departmental Ethics Committee, Department of Zoology, University of Rajasthan, Jaipur, India.

### 2.2. Chemical

Nickel chloride hexahydrate procured from Hi-Media Laboratories Pvt. Ltd., Mumbai, (Purity: 97.0%) was used for the study.

### 2.3. Experimental Design

Animals were divided into four groups of ten mice each. Group I was given tap water and served as a control, while groups II, III, and IV were given 46.125, 92.25, and 184.5 mg Ni/kg b.wt. as NiCl_2_·6H_2_O orally from days 6 to 13 of gestation, that is, organogenetic period. The doses were selected below LD_50_, that is, 369 mg Ni/kg b.wt. Dams were sacrificed by cervical dislocation on day 18 of gestation, and uteri of all the sacrificed dams were examined. Diet consumption, water intake and body weight of all the groups were recorded daily.

### 2.4. Fetal Observations

The implant sites and live fetuses per dam were counted, and the conceptus at each site was classified as being alive, resorbed, or dead. The live fetuses were sexed, weighed, and examined for morphological alterations. Randomly selected 75% of fetuses were fixed in 95% alcohol for double staining (alizarin red S and alcian blue) to observe the skeletal anomalies [18], and the remaining fetuses were fixed in Bouin's fixative to study the brain.

### 2.5. Statistical Analysis

The statistical analysis of the data was evaluated by using the Microsoft Office Excel 2003 software, and the significance of the data was determined either by using one way analysis of variance (ANOVA) or one way Mann-Whitney *U* test. The levels of significance were *P* < 0.05 (almost significant) and *P* < 0.01 (significant).

## 3. Results

The pregnant females administered with Ni^+2^ during organogenetic period revealed an almost significant (*P* < 0.05) decrease in diet consumption after 92.25 mg Ni/kg b.wt. However, after 184.5 mg Ni/kg b.wt., the decrease was significant (*P* < 0.01). Similar pattern of decrease was evident in intake of water. No behavioral as well as morphological changes were evident in the treated dams, although maternal weight decreased significantly (*P* < 0.01) after 92.25 and 184.5 mg Ni/kg b.wt., but no maternal toxicity was observed (Table 1).

**Figure 1:**
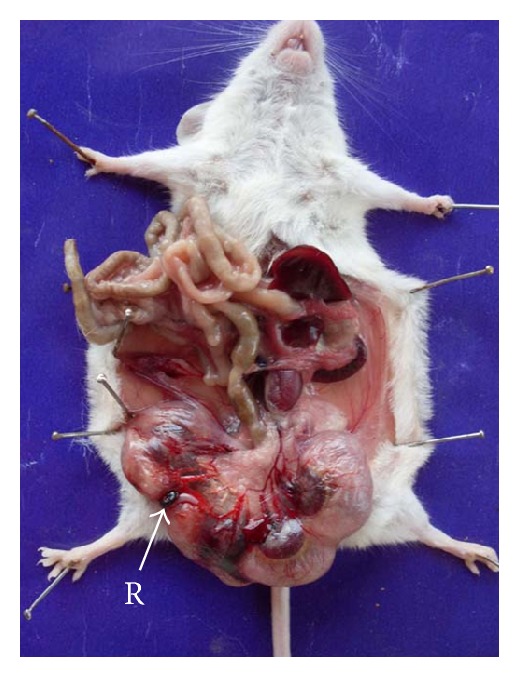
Photograph showing embryonic resorption (R) in left uterine horn at the dose of 46.125 mg Ni/kg b.wt. as NiCl_2_·6H_2_O.

The average implant sites per dam decreased after 92.25 and 184.5 mg Ni/kg b.wt. when compared with the control group. Average number of live fetuses/dam were significantly reduced (*P* < 0.01) in group IV. Further, there was a concomitant rise in the percentage of resorbed, dead, and macerated fetuses. However, in groups II and III, the dead and macerated fetuses were not observed. At 184.5 mg Ni/kg b.wt. dose level, the incidence of postimplantation death (35.29%) was evident. In groups II and III, the loss was 4.16% and 9.09%, respectively (Table 2, Figures 1, 3 and 9).

No change was evident in the sex ratio (M : F) after oral administration of Ni at all the three dose levels. The average fetal weight significantly reduced (*P* < 0.01) in a dose-dependent manner. The placental weight decreased nonsignificantly in all the treated groups (Table 2).

Fetuses with macroscopic anomalies such as open eye lids (10% and 12.50% in groups III and IV) (Figures 4 and 13), club foot (6.25% in group IV) (Figure 13), and umbilical hernia (5% and 6.25% in groups III and IV) (Figures 5 and 12) were evident. Ophthalmic anomalies, namely, microphthalmia (5%, 5%, and 6.25% in all the treated groups) (Figure 11) and exophthalmia (5% in group III) were observed. The occurrence of hydrocephaly in groups III and IV was 5% and 12.50% (Figure 10), and microcephaly in group III was 5%, respectively. No gross anomalies were seen in group II (Table 3).

**Figure 2:**
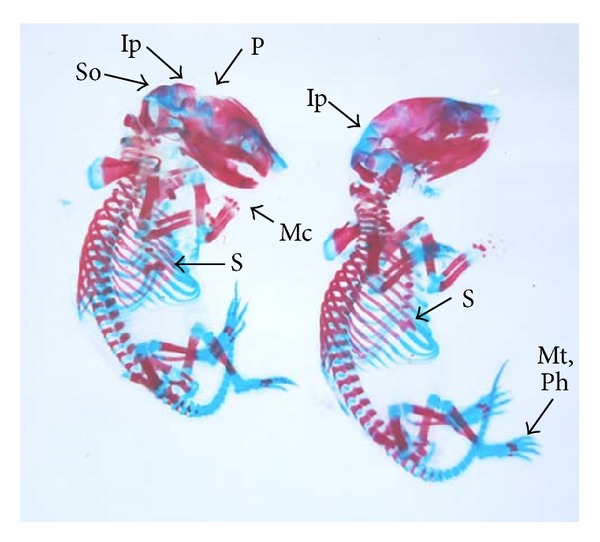
Photograph showing skeleton of fetuses with absence of intraparietal (Ip), reduced ossification of parietal (P) and supraoccipital (So) skull bones, metacarpals (Mc), metatarsals (Mt), phalanges (Ph), and sternebrae were bifurcated (S) (xiphoid) at the dose of 46.125 mg Ni/kg b.wt. as NiCl_2_·6H_2_O.

**Figure 3:**
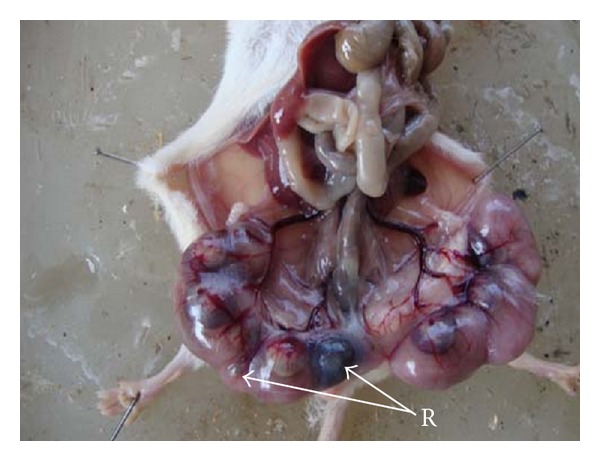
Photograph showing embryonic resorptions (R) in left uterine horn at the dose of 92.25 mg Ni/kg b.wt. as NiCl_2_·6H_2_O.

**Figure 4:**
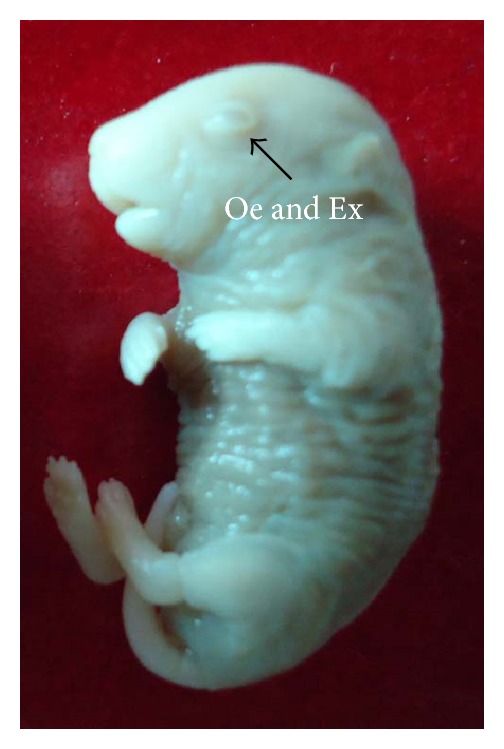
Photograph showing fetus with exophthalmia (Ex) and open eyelid (Oe) at the dose of 92.25 mg Ni/kg b.wt. as NiCl_2_·6H_2_O.

**Figure 5:**
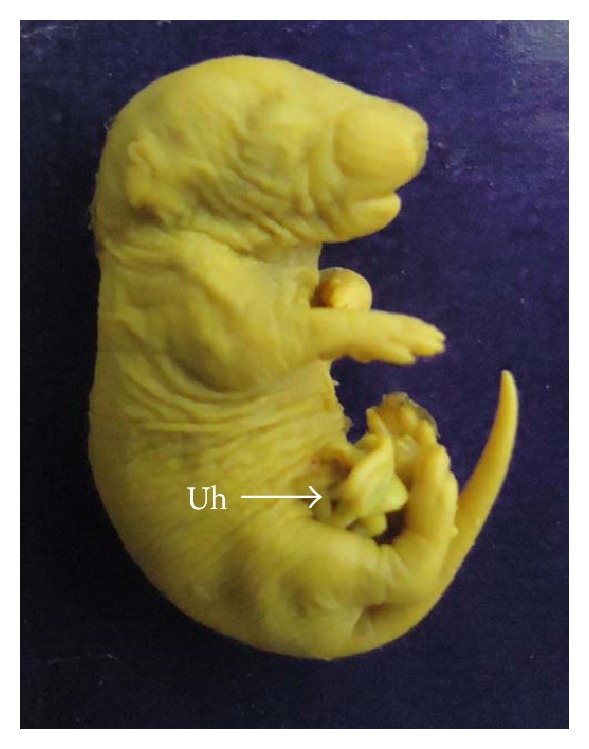
Photograph showing fetus with umbilical hernia (Uh) at the dose of 92.25 mg Ni/kg b.wt. as NiCl_2_·6H_2_O.

**Figure 6:**
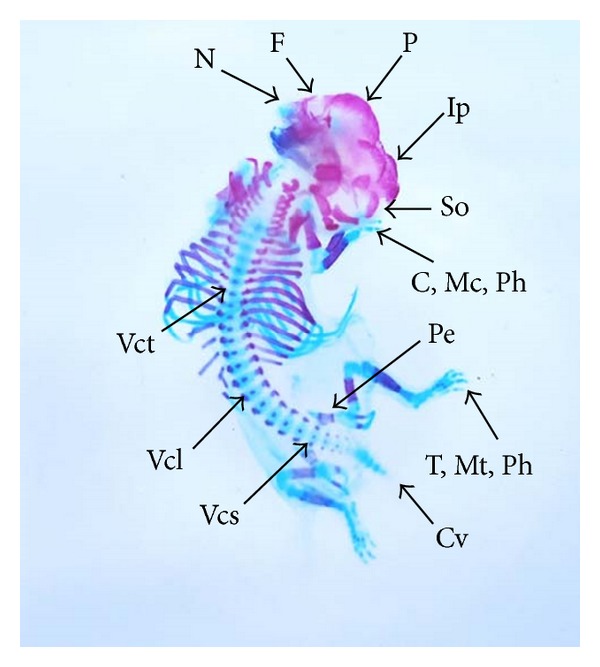
Photograph showing skeleton of fetus with reduced ossification of skull bones—nasal (N), frontal (F), parietal (P), intraparietal (Ip) and supraoccipital (So), vertebral centra (Vc) in thoracic (Vct), lumbar (Vcl) and sacral (Vcs) regions, and pelvic elements (Pe). Note the complete absence of carpals (C), metacarpals (Mc), tarsals (T), metatarsals (Mt), phalanges (Ph), and caudal vertebrae (Cv) at the dose of 92.25 mg Ni/kg b.wt. as NiCl_2_·6H_2_O.

**Figure 7:**
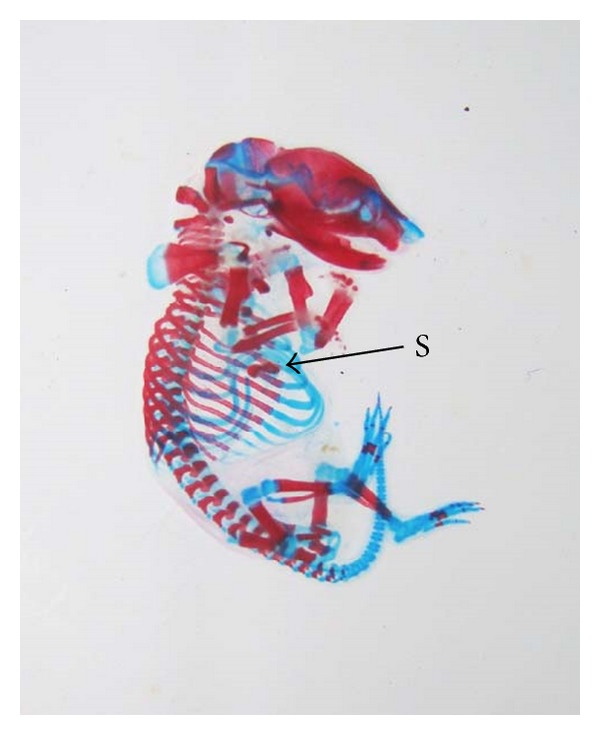
Photograph showing skeleton of fetus with bifurcate sternebrae (S) (xiphoid) at the dose of 92.25 mg Ni/kg b.wt. as NiCl_2_·6H_2_O.

**Figure 8:**
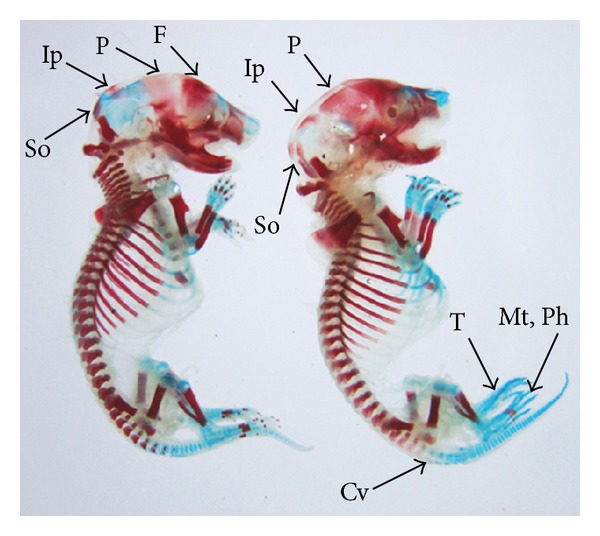
Photograph showing skeleton of fetuses with reduced ossification of frontal (F), parietal (P), intraparietal (Ip), supraoccipital (So) skull bones, and caudal vertebrae (Cv). Absence of tarsals (T), metatarsals (Mt), and phalanges (Ph) at the dose of 92.25 mg Ni/kg b.wt. as NiCl_2_·6H_2_O.

**Figure 9:**
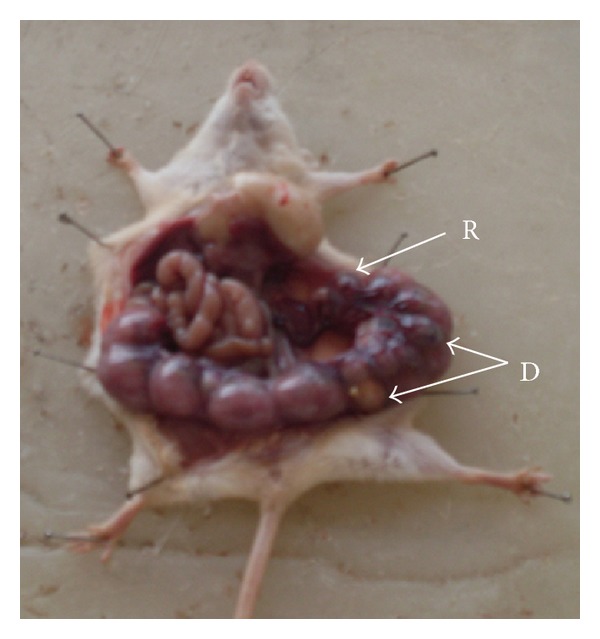
Photograph showing embryonic resorptions (R) and dead (D) fetus in right uterine horn at the dose of 184.5 mg Ni/kg b.wt. as NiCl_2_·6H_2_O.

**Figure 10:**
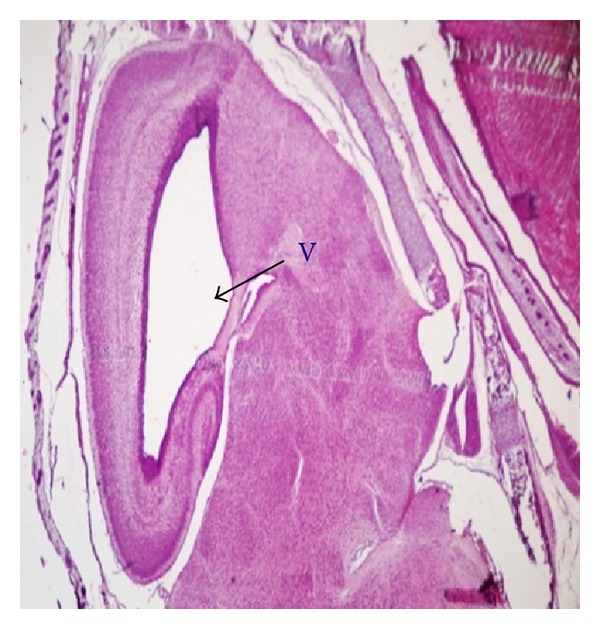
Microphotograph of sagittal section of the fetal brain exhibiting hydrocephaly, that is, enlargement of ventricle (V) at the dose of 184.5 mg Ni/kg b.wt. as NiCl_2_·6H_2_O.

**Figure 11:**
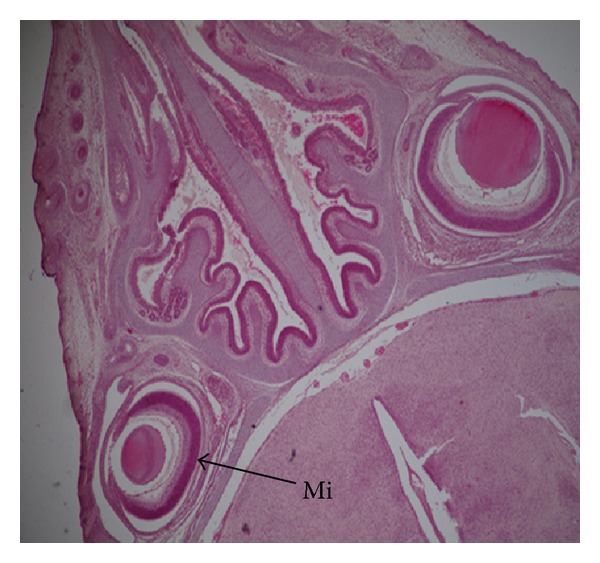
Microphotograph of transverse section of the fetus brain revealing unilateral microphthalmia (M) at the dose of 184.5 mg Ni/kg b.wt. as NiCl_2_·6H_2_O.

**Figure 12:**
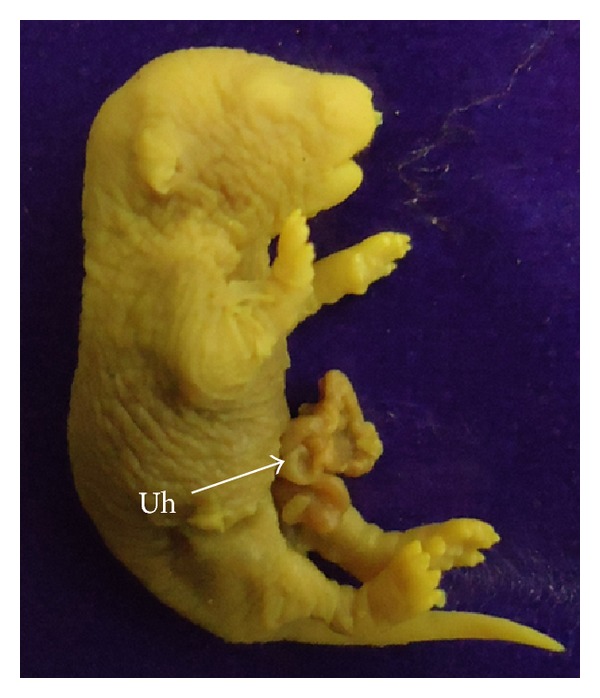
Photograph showing fetus with umbilical hernia (Uh) at the dose of 184.5 mg Ni/kg b.wt. as NiCl_2_·6H_2_O.

**Figure 13:**
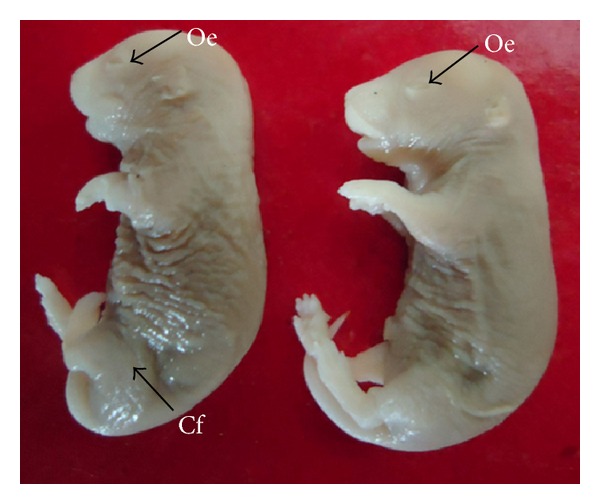
Photograph showing fetuses with open eyelids (Oe) and club foot (Cf) at the dose of 184.5 mg Ni/kg b.wt. as NiCl_2_·6H_2_O.

**Figure 14:**
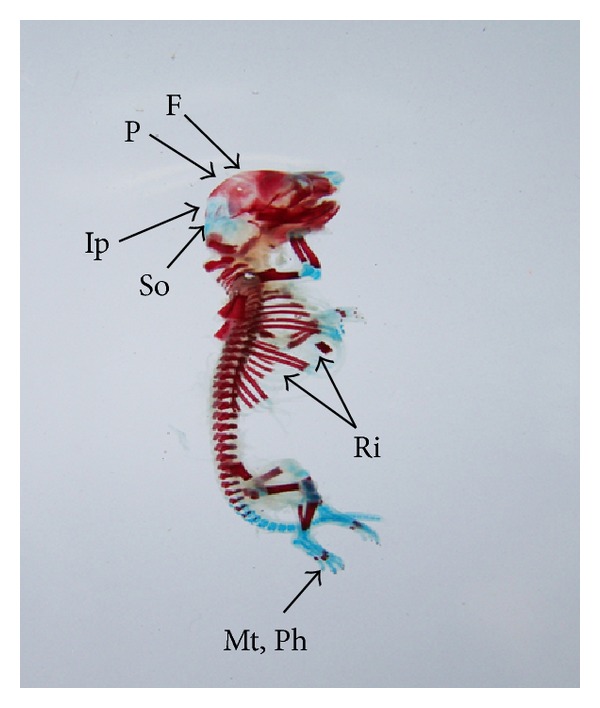
Photograph showing skeleton of fetus with reduced ossification of skull bones—frontal (F), parietal (P), intraparietal (Ip) and supraoccipital (So), gap between the ribs (Ri) and reduced ossified metatarsals (Mt), and phalanges (Ph) at the dose of 184.5 mg Ni/kg b.wt. as NiCl_2_·6H_2_O.

**Figure 15:**
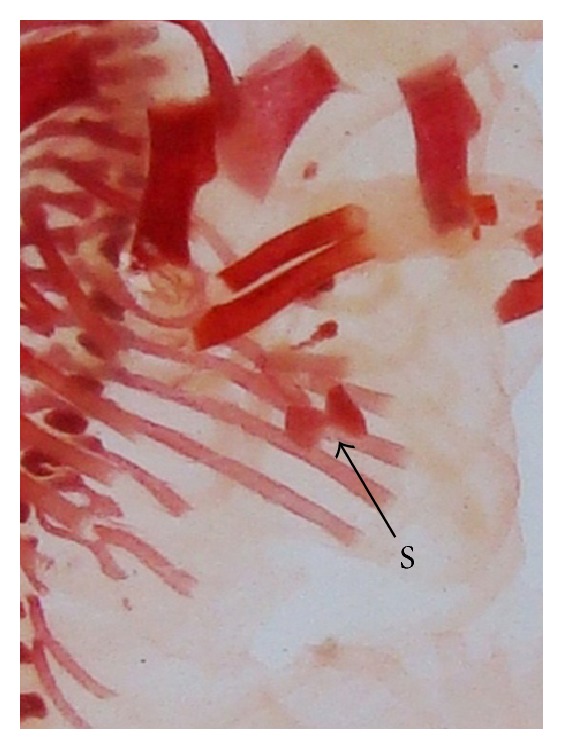
Photograph showing skeleton of fetus with bifurcate sternebrae (S) (Xiphoid) at the dose of 184.5 mg Ni/kg b.wt. as NiCl_2_·6H_2_O.

**Figure 16:**
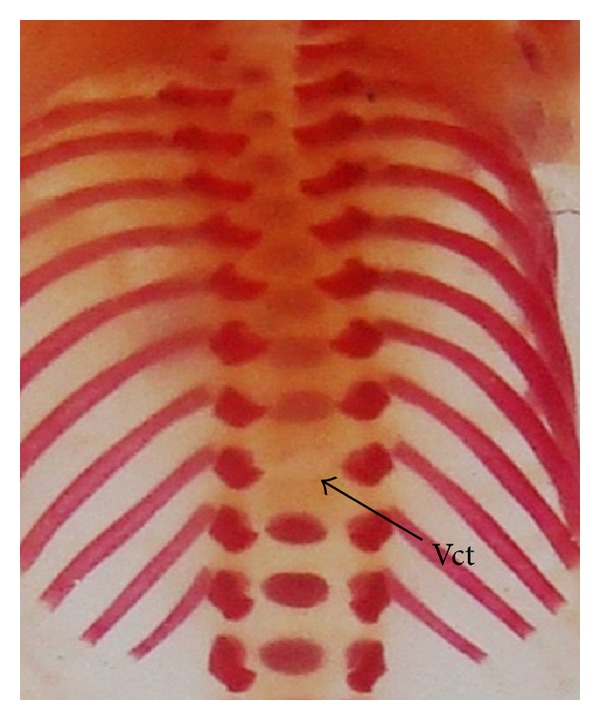
Photograph showing skeleton of fetus with absence of vertebral centra in thoracic region (Vct) at the dose of 184.5 mg Ni/kg b.wt. as NiCl_2_·6H_2_O.

**Figure 17:**
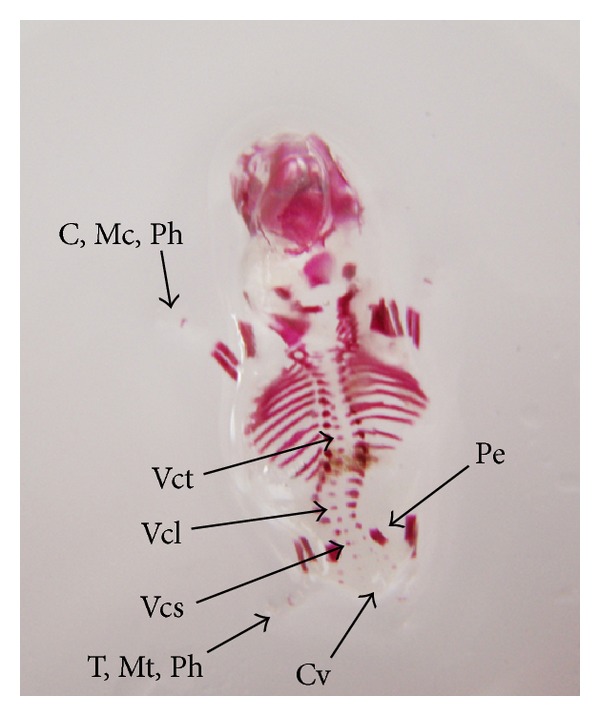
Photograph showing skeleton of fetus with incomplete ossification of vertebral centra (Vc) in thoracic (Vct), lumbar (Vcl) and sacral (Vcs) regions, and pelvic elements (Pe). Note the complete absence of carpals (C), metacarpals (Mc), tarsals (T), metatarsals (Mt), phalanges (Ph), and caudal vertebrae (Cv) at the dose of 184.5 mg Ni/kg b.wt. as NiCl_2_·6H_2_O.

The double stained (alizarin red S and alcian blue) skeleton of fetuses showed numerous anomalies such as reduced ossification of nasal, frontal, parietal, intraparietal, and supraoccipital bones of skull, absence or gap between the ribs, reduced or fused sternebrae, reduced/absence/displaced vertebral centra in thoracic and lumbar regions, reduced/absence of caudal vertebrae, reduced pelvic elements, and absence of carpals, metacarpals, tarsals, metatarsals, and phalanges. The percentage of skeletal anomalies was found to be dose dependent (Table 4, Figures 2, 6, 7, 8, 14, 15, 16, and 17).

## 4. Discussion

Administration of nickel as nickel chloride hexahydrate via oral intubation during the period of organogenesis exhibited decrease in diet consumption, water intake, and weight of the pregnant mice. Fetal development retardation and embryo fetotoxicity were observed as evident by the reduction in fetal and placental weights, number of live fetuses, and higher incidences of resorptions and postimplantation death. Exposure also increased the fetal as well as skeletal anomalies.

A significant reduction in maternal weight was observed in the treated group. No mortality occurred in any experimental groups. This decrease in maternal weight can be correlated to decrease in diet consumption and water intake. Adjroud [8] also observed similar phenomena. However, this alone cannot contribute to decreased maternal weight. It could also possibly be due to increased degeneration of lipids and proteins leading to decreased organ weight as a result of nickel toxicity [19, 20]. Other factors may also be responsible for the reduced maternal body weight, which could probably be due to NiCl_2_-induced resorptions, decreased weight, and growth of the fetus. The average number of live fetuses per dam was another parameter, which was adversely affected after NiCl_2_ administration. A concurrent rise in the number of resorbed and dead embryos/fetuses along with reduced number of live fetuses per dam was observed. Thus, the evident decline in the number of live fetuses per dam is probably due to increased percentage of resorbed embryos. Similar results were also obtained by several authors [16, 17, 21], who observed a decrease in number of live fetuses due to an increased number of resorptions in mice and rats.

High incidences of postimplantation death were observed in a dose-dependent manner either as embryonic resorption or death. The toxic effects of Ni^+2^ have been shown to occur in mouse embryos during the passage through the oviduct [22] and during organogenesis [23] with subsequent cytotoxic and teratogenic effects occurring after implantation. Soluble NiCl_2_ has been observed in the cytoplasm and nucleus [24], and Chen et al. [25] hypothesized that divalent metal transporter-I (DMT-I) is utilized by nickel ions to enter the cells. Excess of nickel ions may therefore replace the other metal ions that are required for the structure and function of enzymes leading to their inactivation [24], which would contribute to nickel induced embryo and fetotoxic effects. Embryotoxicity and fetotoxicity of nickel has been observed in mice, rats, and women by several authors [7–12, 16].

A dose-dependent decline in fetal weight was observed in all the experimental groups. This correlates well with the decrease in maternal weight. A similar relationship was observed by other environmental contaminants such as chromium in rats [26] and cadmium in mice [27]. Decrease in fetal body weight is a sensitive and precise indicator for growth retardation. The plausible cause of such an association may be due to maternal organism being under stress, which in turn might affect the growing fetuses leading to its growth retardation and hence reduced weight of the fetuses. New and Coppola [28] reported that acute interruption of blood flow to the uterine horn caused growth retardation of the fetuses. Reduction in the uterine vascularization subsequently decreases blood flow to the uterine horn and this induces fetal-placental growth retardation [29]. The reduced weight of the fetuses was accompanied by incomplete ossification of the fetal skeleton, which may be one of the factors for growth retardation leading to reduced weight of the fetuses. Similar relationship between decrease in body weight of the fetuses and retarded ossification of the skeleton has been reported [16, 17, 21].

Brain anomalies such as microcephaly and hydrocephaly have also been observed in the present study. Exposure to methyl mercury leads to disturbances in the development of the brain, which included microcephaly caused due to dilation of lateral ventricles and derangement in the fundamental structure of gray matter as a result of abnormal neuronal migration [30]. Enlargement of cerebral ventricles (hydrocephaly) have also been reported in various metals like gold [31], chromium [32], and mercury [33]. The speculated cause of such abnormality is fluid retention in the brain, which resulted in enlargement of the ventricles. The ependymal cells and choroid plexus epithelial cells observed after mercury treatment retained large amount of mercury within their cytoplasm [34], and this may have contributed to the development of hydrocephaly either by (a) causing disturbances in the cerebrospinal fluid (CSF) [35] or (b) changes in CSF content including reduced proteoglycan detectable prior to morphological brain defects [36], which probably may be responsible for enlargement of ventricle structures.

The occurrence of open eyelids in the present study were noted in few fetuses after nickel chloride administration, which probably may be due to partial ossification of skull bones that may have affected the diameter of eye orbit. This could result in changes in the attachment of eye muscles thereby leading to the condition of open eyes. After exposure with environmental contaminants such as mercury and lead similar anomalies were also reported [37, 38]. Ophthalmic malformations such as microphthalmia and exophthalmia have been observed after nickel chloride administration in few fetuses in the present study. Higher incidences of ocular anomalies in rats such as microphthalmia and anophthalmia have been reported on exposure of nickel carbonyl by inhalation [39]. The retina of microphthalmic eyes was unusually thickened, folded, or redundant. There was reduced thickness of the neuroblastic layer, necrosis, or pyknosis of retinal cells and attenuation of the optic nerve [39].

Occurrence of umbilical hernia—an outward bulging or protrusion of the part of the abdominal organs was observed in the experimental groups. This is in concomitance with the results obtained by Szabo et al. [31] who noticed umbilical hernia with protrusion of the intestinal loops after administration of gold in rats and cadmium in hamsters [40]. Further, deformities in limb such as club foot were evident after NiCl_2_ administration. Club foot formation can possibly be due to (i) indirect action of metal, (ii) alteration of maternal physiology, which disturbs the hormonal balance in mother, or (iii) direct effect on the tissue primordial of foot [38]. Similar were the observations with lead [41] and mercury [42].

Double stained skeletons of exposed fetuses revealed reduced ossification of skull bones (nasal, frontal, parietal, intraparietal, and supraoccipital), reduced/wavy ribs, reduced/bifurcate sternebrae, reduced number of pelvic elements, reduced ossification/absence of carpals, metacarpals, tarsals, metatarsals, phalanges, and caudal vertebrae, and displaced/absence of vertebral centra in thoracic, lumbar, and sacral regions. Exposure to various other metals like chromium [26], cadmium [40, 43, 44], and gold [31] has also been reported to produce such effects. This reduced ossification of various bones may be due to altered calcium metabolism or decreased calcium and magnesium ion levels as well as altered calcitonin level in the growing fetus, thereby causing retardation in bone development.* In vitro* study revealed that osteoblasts exposed to Ni^+2^ showed significant decreases in alkaline phosphatase activity [45]. Osteoblasts cultured with NiCl_2_ resulted in severe osteoblast apoptosis [46] and dysfunction [47]. Administration of Ni^+2^ in rats resulted in a decreased number of primary and secondary osteons [48].

## 5. Conclusion

Conclusively, the present investigations with nickel exhibited no maternotoxicity in terms of mortality but demonstrated significant reduction in weight of the dams. Further, it elicited fetotoxicity and teratogenicity as seen in the terms of reduced number of implant sites and average number of live fetuses/dam, reduced fetal and placental weights, and increased percentage of postimplantation death and various terata. This reflects that prenatal exposure of nickel affects maternal physiology leading to vulnerability of the fetus as nickel appears to cross the placental barrier which poses a risk to the developing fetuses.

## Figures and Tables

**Table 1:** Effect of Ni^+2^ (as NiCl_2_·6H_2_O) on diet consumption, water intake, and weight gain of pregnant mice.

Parameters	Group I (control)	Group II (46.125 mg Ni/kg b.wt.)	Group III (92.25 mg Ni/kg b.wt.)	Group IV (184.5 mg Ni/kg b.wt.)
	(Mean ± SEM)			
Diet consumption (gm)	8.11 ± 0.16	8.42 ± 0.60	7.00 ± 0.00*	6.11 ± 0.01**
Water intake (mL)	11.00 ± 0.00	11.88 ± 0.00	10.86 ± 0.26*	9.62 ± 0.09**
Maternal weight (gm)	16.6 ± 0.60	15.00 ± 0.57	12.00 ± 0.00**	9.60 ± 0.74**
Percent mortality	0	0	0	0

One way analysis of variance.

*Almost significant (*P* < 0.05).

**Significant (*P* < 0.01).

**Table 2:** Influence of Ni^+2^ (as NiCl_2_·6H_2_O) on embryonic developmental of mice.

Parameters	Group I (control)	Group II (46.125 mg Ni/kg b.wt.)	Group III (92.25 mg Ni/kg b.wt.)	Group IV (184.5 mg Ni/kg b.wt.)
Average number of implant sites/dam (I)	8.00 ± 0.57	8.00 ± 0.57	7.33 ± 0.33	6.80 ± 1.49
Average number of live fetuses/dam (II)	8.00 ± 0.57	7.66 ± 0.66	6.66 ± 0.33	4.40 ± 0.87**
Resorbed embryos (%)	—	4.16	9.09	23.52
Dead fetuses (%)	—	—	—	5.88
Dead macerated fetuses (%)	—	—	—	5.88
Postimplantation death (%)	—	4.16	9.09	35.29
Sex ratio (M : F)	54.16 : 45.83	52.63 : 47.36	50.00 : 50.00	52.94 : 47.05
Fetal weight (gm) (III)	1.35 ± 0.00	1.29 ± 0.018*	1.18 ± 0.022**	0.82 ± 0.063**
Placental weight (gm) (IV)	0.12 ± 0.002	0.10 ± 0.003	0.10 ± 0.004	0.09 ± 0.004

(I) and (II) analyzed by one way Mann Whitney *U*-test.

(III) and (IV) analyzed by one way analysis of variance.

*Almost significant (*P* < 0.05).

**Significant (*P* < 0.01).

**Table 3:** Fetuses with different anomalies after maternal exposure to Ni^+2^ (as NiCl_2_·6H_2_O).

Parameters (%)	Group I (control)	Group II (46.125 mg Ni/kg b.wt.)	Group III (92.25 mg Ni/kg b.wt.)	Group IV (184.5 mg Ni/kg b.wt.)
Hydrocephaly	0	0	5.00	12.50
Microcephaly	0	0	5.00	0
Open eyelids	0	0	10.00	12.50
Microphthalmia	0	5.00	5.00	6.25
Exophthalmia	0	0	5.00	0
Anophthalmia	0	0	0	0
Club foot	0	0	0	6.25
Umbilical hernia	0	0	5.00	6.25

**Table 4:** Fetuses with skeleton anomalies after maternal exposure to Ni^+2^ (as NiCl_2_·6H_2_O).

Parameters (%)	Group I (control)	Group II (46.125 mg Ni/kg b.wt.)	Group III (92.25 mg Ni/kg b.wt.)	Group IV (184.5 mg Ni/kg b.wt.)
Total fetuses with different skeletal anomalies	0	22.7	35.0	50.0
Skull	0	18.1	25.0	40.6
Vertebral column	0	0	5.0	9.3
Ribs	0	4.5	5.0	6.2
Sternum	0	9.0	15.0	18.7
Pelvic elements	0	0	5.0	6.2
Caudal vertebrae	0	0	5.0	6.2
Carpals, metacarpals, tarsals, metatarsals, and phalanges	0	18.1	25.0	46.8
